# Microencapsulation by Spray Drying of Bioactive Compounds: A Comparison Between Pulp or Acidified Extract of Jussara Fruit (*Euterpe edulis* Martius)

**DOI:** 10.3390/plants14091295

**Published:** 2025-04-25

**Authors:** Isabela Carolina Ferreira da Silva, Silvio Claudio da Costa, Grasiele Scaramal Madrona, Rita de Cássia Bergamasco

**Affiliations:** 1 Postgraduate Program in Food Engineering, Universidade Estadual de Maringá, Avenida Colombo 5790—Zona 7, Maringá 87020-900, PR, Brazil; isabelacfes@gmail.com (I.C.F.d.S.); gsmadrona@uem.br (G.S.M.); 2 Biochemistry Department, State University of Maringá (UEM), Avenida Colombo 5790—Zona 7, Maringá 87020-900, PR, Brazil; sccosta@uem.br; 3 Food Engineering Department, State Universidade Estadual de Maringá, Avenida Colombo 5790—Zona 7, Maringá 87020-900, PR, Brazil

**Keywords:** anthocyanins, color stability, natural colorant, microencapsulation

## Abstract

The present study aims to evaluate the best way to use the fruit of the Jussara Palm (*Euterpe edulis* Martius), whether as pulp or acidified extract, in microencapsulation by spray drying, to develop a natural dye with attractive, stable color characteristics, and high anthocyanin content. The anthocyanins were extracted using a hydroalcoholic solution (70%) acidified with citric acid, and the microcapsules were produced in a proportion of in a 1:3 ratio (pulp/extract: agent), using maltodextrin and gum arabic as encapsulating agents. The microcapsule samples showed high values of encapsulation efficiency and retention of total anthocyanins, with the pulp showing higher levels of bioactive compounds compared to the extract. Regarding characterization, the microcapsules obtained from pulp showed better results for total anthocyanins (77.5 mg cyanidin-3-O-glucoside/100 g), phenolic compounds (492.48 mg EAG/100 g) and antioxidant activity (85.82%). The best color stability, with storage at room temperature, was also observed in microcapsules obtained from Jussara pulp (ΔE 6.25). Thus, the pulp presents better technological characteristics for microcapsule preparation by spray drying, with potential application as a natural dye in food matrices.

## 1. Introduction

The fruit of the Jussara Palm tree (*Euterpe edulis* Martius) has aroused interest, both in science and industry, mainly because it is rich in bioactive compounds, such as anthocyanins, the main compounds studied in the fruit. The extraction of these bioactive compounds is an essential technique for maintaining the stability of these substances, which are highly valued for their antioxidant properties and potential health benefits. Recent research has shown that the use of hydroalcoholic solutions, acidified or not, are effective methods for maximizing the extraction of these compounds. These studies show that the combination of solvents and pH adjustments can significantly improve the efficiency of the extraction process, while preserving the integrity and bioactivity of Jussara anthocyanins [1]. Thus, the compounds of this fruit have been successfully applied in food matrices, including in microencapsulated form, which guarantees their greater stability during storage.

The spray-drying microencapsulation technique is widely recognized for its efficiency in stabilizing bioactive compounds. This method protects the compounds from unwanted physical and chemical interactions with the external environment, resulting in a prolonged shelf life and expanding their possibilities for industrial application [2]. The combination of encapsulating agents, such as gum arabic and maltodextrin, is widely used for encapsulating anthocyanins due to their excellent stabilizing properties. These characteristics protect the bioactive compounds from thermal degradation during the spray-drying process and during storage, forming complexes that improve their stability [3]. In addition, gum arabic stands out for its high solubility in water and its remarkable flavor-retention capacity, which significantly improves the sensory properties of the final product, with the aim of subsequent application in food matrices. Therefore, the spray-drying technique is an excellent alternative for processing the fruit of the Jussara Palm. The resulting powder is significantly easier to handle, package and transport compared to fresh fruit. In addition, this technique guarantees a high quality product with an extended shelf life. [4].

The literature reports several studies on microencapsulation of bioactive compounds from Jussara fruit, using the spray-drying technique. In these works, the fruit is used both in the form of pulp [5,6,7,8] and extract [9,10,11,12]. However, research is limited when it comes to comparisons between the pulp and the acidified extract of this fruit. This study stands out precisely because it fills this gap, offering an analysis of the differences and similarities in microencapsulation efficiency and the stability of bioactive compounds, especially due to the way they are used in microencapsulation, which can influence the characteristics of the microcapsules, as well as the retention of bioactive compounds. Investigating these variations is crucial in order to better understand how to optimize the use of these compounds in different food matrices and expand the potential for commercial applications.

Therefore, the present study aims to evaluate the best way to use Jussara fruit (pulp or acidified extract) in microencapsulation by spray drying, with the aim of developing a natural dye with stable attractive color characteristics and high anthocyanin content.

## 2. Results and Discussion

### 2.1. Characterization of the Pulp and Acidified Extract

The content of total monomeric anthocyanins (TA) present in the pulp is 2.31 times greater than the content of anthocyanins present in the acidified extract. Regarding total phenolic compounds, this value is 1.76 times higher in the pulp (Table 1).

Paim et al. [7] reported anthocyanin concentrations of 104.66 ± 2.70 mg cyanidin-3-glucoside/100 g for whole pulp and 89.10 ± 1.82 mg cyanidin-3-glucoside/100 g for centrifuged pulp. These values are very similar to those found in the present study, which also subjected the pulp to the centrifugation process. Centrifugation can significantly influence the concentration of bioactive compounds by separating suspended solids, resulting in a purer extract free of impurities, which improves the stability of the compounds and the effectiveness of the final extract. This process facilitates homogenization and subsequent spray drying, providing cleaner samples and more accurate analysis readings. In our study, the pulp was centrifuged at 10 °C for 15 min at 8000 rpm, ensuring greater purity of the extract and improving the accuracy of subsequent analyses.

The concentration of anthocyanins and phenolic compounds in the extract is directly linked to the degree of extraction, which is influenced by various factors, such as the proportion between solvent and sample mass, the type of solvent chosen, as well as the pH and temperature of the process [13]. As it is a solution containing only 28% pulp after concentration, it appears that the extraction process was efficient, and the sample is an excellent source of bioactive compounds.

Through the method of elimination of DPPH radicals (%), the antioxidant capacity is directly linked to the concentration of phenolic compounds present in the fruits [14]. The pulp presented greater antioxidant capacity than the extract, which was an expected result taking into account the higher phenolic content present in the pulp compared to the extract.

### 2.2. Microcapsule Characterization

The microcapsule samples showed statistical significance at the 5% level, for content of total anthocyanins (TAs) and phenolic compounds (CFTs) (Table 2).

When comparing with results in Table 1, characterizing pulp and acidified extract, it was expected that the microcapsules would follow the same behavior in relation to levels of these bioactive compounds, that is, the microcapsules obtained from pulp (MP) presented greater results compared to the acidified extract (ME). This difference is due to the relationship between the solvent and sample mass used during extraction. In addition, it is important to consider that the decrease in bioactive compounds in microcapsules is associated with the sensitivity of these compounds to light, temperature and oxygen. During the spray-drying process, where the solutions are exposed to a temperature of 175 °C, these factors may have contributed to a reduction in their stability and content.

The microencapsulated phenolic compounds were able to scavenge the stable DPPH radical, and the microencapsulation process preserved pulp antioxidant activity. However, the extract showed a significant decrease in antioxidant capacity, which may be related to the drying process where a 25.96% reduction in phenolic compounds occurred.

Regarding encapsulation efficiency (EE), although the microcapsules produced from pulp have a higher anthocyanin content, statistically there was no significant difference (*p* < 0.05) between samples. It is worth noting that encapsulation efficiencies are also related to the stability of anthocyanins and phenolic compounds. Consequently, the results obtained indicate that the combination of maltodextrin and gum arabic formed suitable chemical interactions in which they were able to retain the monomeric anthocyanins and phenolic compounds during the spray-drying process [15].

The samples showed a high retention index (RTA), which indicates that the spray-drying process is effective for obtaining microencapsulated anthocyanins, despite being a technique that requires high temperatures, a factor considered harmful to bioactive compounds.

### 2.3. Color Stability

The color parameters were used to verify sample color stability during 100 days of storage at room temperature, and the results are presented in Table 3.

**Figure 1:**
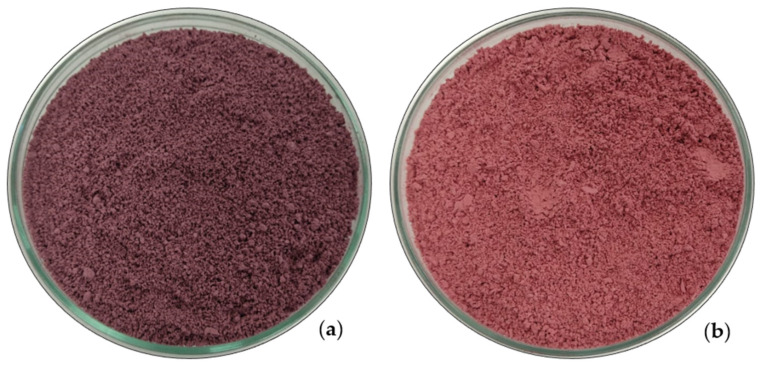
Microencapsulated pulp (**a**) and microencapsulated acidified extract (**b**).

ME presented higher luminosity values (L*), indicating a lighter color, which was already expected as it has a pink color compared to the pulp, which has a purple color (Figure 1). The color of anthocyanins is directly related to the pH of the medium. At acidic pH, from 1 to 3, as is the case of ME, anthocyanins show a more intense red color; and as the pH increases (>4.5), as in the case of MP, the predominant color becomes blue [16].

In relation to the a* parameter, ME tends towards red and MP is closer to green. For the b* parameter, it is possible to notice that MP presents a color tending to blue; in contrast ME tends towards yellow. According to Souza et al. [17], the increase in blue color may be related to a reduction in the anthocyanins that give this hue. Consequently, the sample starts to contain a greater quantity of red anthocyanins, increasing the a* parameter. It is possible to verify this statement in the results, where MP starts to show a decrease in parameter b* and increase in a* during storage time. Although ME presents a color tending to yellow, the statement also applies to this sample, as a decrease in b* is observed.

Chromaticity (C*) indicates color intensity, therefore, the ME presents a higher concentration of color compared to the pulp, due to its saturation. With increasing chromaticity, extract color becomes more vivid/intense, as MP is less saturated, presenting a more opaque color. The hue angle (h°) is attributed to the perception of colors such as red, green and blue [18]. Over time, an increase in h° values for MP is observed, causing an increase in the perception of the bluish red color. In relation to the ME, the red color became more noticeable, with a decrease in the tone angle.

Throughout the storage period, both samples showed changes in color. However, this change was smaller in the microcapsules produced from pulp (lower ΔE*), indicating that this sample was more stable during storage, maintaining its color, a relevant factor for subsequent application as a dye.

Although it is clear that MP presents better results, ME is also an excellent source of bioactive compounds, in addition to its use presenting some advantages: as the fruit of the Jussara Palm corresponds to only 10% pulp, a smaller amount of material would be needed for obtaining anthocyanins. Also, the acidified extract is an easy-to-handle material during the spray-drying microencapsulation process, compared to the pulp, which contains a higher amount of suspended solids and can cause problems with the equipment.

## 3. Materials and Methods

### 3.1. Processing of Fruit

The fruits of the Jussara Palm (*Euterpe edulis* Martius) were harvested in the city of Jandaia do Sul, Paraná in June 2022. After harvesting, the fruits were selected according to their stage of ripeness (ripe and purple), washed and sanitized with sodium hypochlorite solution. The pulping process began by immersing the fruit in drinking water heated to 40 °C for 30 min, softening the peel. Water was then added at a rate of 0.3 L/kg to the softened fruit, which was then manually macerated and the various fruit fractions (peel, seed and pulp) separated. The pulp obtained was stored frozen and then thawed according to the quantity required for each analysis.

### 3.2. Acidified Extract with Citric Acid

For the extraction of anthocyanins, the methodology proposed by Lima et al. (2019) [19] was followed, with modifications. The extract was prepared at a concentration of 25 g/100 mL of hydroalcoholic solution of 70%, using water acidified with citric acid to pH 2.0, since the acidification process helps maintain the stability of the anthocyanins [6]. The extraction took place in the absence of light, under refrigeration at 8 °C ± 2 °C for 24 h to avoid degradation of the compounds present. Subsequently, the extract was centrifuged in a Hettich Universal model 320R centrifuge, at 10 °C for 15 min and 8000 rpm, to reduce the amount of suspended solids. The extract was then concentrated in a Tecnal model TE-211 rotary evaporator at 45 °C, presenting a final concentration of 28% pulp.

### 3.3. Processing of Microcapsules

The processing of microcapsules followed the methodology proposed by Lima et al. (2019) [19], with modifications. First, the pulp was centrifuged at 10 °C for 15 min and 8000 rpm, to reduce suspended solids. A solution of the encapsulating agents maltodextrin and gum arabic (30% *w*/*v*) was added to the pulp/extract in a ratio of 1:3 (pulp/extract:agent), and kept under stirring at 150 rpm in a mechanical stirrer (Fisatom, Model 713) until complete dissolution, at room temperature. For microencapsulation, the solutions were dried in a BUCHI model B-191 spray dryer, under the following drying conditions: nozzle diameter of 7 mm, drying air inlet temperature of 175 °C and outlet of 100 °C, rate of 16% peristaltic pump (4.44 mL/min), 100% aspiration rate and average feed flow of 500 mL/h. The dry material obtained was stored in airtight bottles and protected from light. The microcapsules produced from the pulp were called MP, and microcapsules produced from extract were called ME.

### 3.4. Analysis of Bioactive Compounds

#### 3.4.1. Determination of Total Anthocyanins

Total monomeric anthocyanins were determined by the differential pH spectrophotometric method, following the methodology described by Giusti and Wrolstad [20]. The absorbance was checked in a spectrophotometer (Bel UV-Vis, model Uv-m51) at 520 and 700 nm after 20 min of incubation at 25 °C. The results were expressed as mg of cyanidin-3-O-glucoside per 100 g of sample.

#### 3.4.2. Phenolic Compounds

The content of total phenolic compounds was determined by the Folin-Ciocalteau spectrophotometric method, following the methodology described by Singleton et al. [21]. The absorbance was checked in a spectrophotometer (Bel UV-Vis, model Uv-m51) at 725 nm after 30 min of incubation at 25 °C. A calibration curve was prepared, using gallic acid as standard, with concentrations between 0.001 and 1 mg/mL. The results were expressed in mg of EAG/100 g of sample.

#### 3.4.3. Total Antioxidant Activity by the DPPH Method

The antioxidant activity was determined by the DPPH radical scavenging method, according to the methodology described by Brand-Williams et al. [22]. The absorbance was checked in a spectrophotometer (Bel UV-Vis, model Uv-m51) at 517 nm. The results were expressed in terms of free radical scavenging (%).

### 3.5. Encapsulation Efficiency (%)

The encapsulation efficiency (EE%) was determined using the total anthocyanin content (AT) and the internal anthocyanin content (IA) present in the microcapsules using the differential pH method, following the methodology proposed by Xue et al. [3]. The AT was determined according to the methodology described in item 3.4.1, while to determine the internal content about 1 g of microcapsules were washed with ethanol using constant agitation in shaker (Marconi model MA 420) for 30 min at 25 °C, to remove the anthocyanins present on the surface of the microcapsules. The material obtained was then filtered and dried at 40 °C. The dried material was then dissolved in distilled water and its AI content determined. The encapsulation efficiency (EE%) was determined according to the following equation:

EE% = IA/AT

### 3.6. Retention (%)

The retention content of total anthocyanins (RTA%), after the spray-drying process was determined according to the methodology of Santana et al. [4], using differential pH. The RTA% content was determined according to the following equation:

RTA% = (ATpowder/ATpulp) × 100

where ATpowder is the anthocyanin content present in the microcapsules and ATpulp is the content present in the pulp of the Jussara Palm fruit.

### 3.7. Color Stability

The instrumental color of the microcapsules was measured using a Chroma meter CR-400/410 colorimeter. The CIEL*a*b* system was applied to describe the color variation of the anthocyanins, the color measurements were expressed in terms of L* (luminosity), a* (red-green), b* (yellow-blue), C* (chroma indicating color intensity) and h (color tone angle).

The color stability of the samples during storage at room temperature was measured by means of instrumental color, after drying in a spray dryer, and after 100 days of storage, to check for color changes over time. The color difference (ΔE*) was calculated according to the equation proposed by Bernardes et al., 2019 [9]:

$${\Delta E}^{*}={\left[{\left({\Delta L}^{*}\right)}^{2}+{\left({\Delta a}^{*}\right)}^{2}+{\left({\Delta b}^{*}\right)}^{2}\right]}^{\frac{1}{2}}$$

### 3.8. Statistical Analysis

All experiments were carried out in triplicate and the results are expressed as mean ± SD (standard deviation). The data collected during the experiment were submitted to analysis of variance (ANOVA) to determine whether there were statistically significant differences between them by comparing means, using SISVAR software (version 5.6). Significant differences were calculated using Tukey’s test at a 5% significance level.

## 4. Conclusions

The fruit of the Jussara Palm tree is an excellent source of bioactive compounds, whether as pulp or acidified extract; however, as it contains a higher concentration of bioactive compounds, the pulp showed better results in terms of anthocyanins, phenolic compounds and antioxidant activity, in microencapsulation by spray drying. When evaluating color stability during storage, better results were also observed for microcapsules produced from pulp, with less color variation over time. Therefore, the pulp presents better technological characteristics and color stability during storage when preparing microcapsules using the spray-drying technique.

## Figures and Tables

**Table 1:** Total anthocyanins (TA), phenolic compounds (CFT) and DPPH antioxidant activity of the pulp and extract of the Jussara fruit.

Treatments		Parameters	
	AT (mg/100 g)	CFT (mg/100 g)	DPPH (%)
Pulp	90.10 ^a^ ± 2.39	626.50 ^a^ ± 2.15	83.59 ^a^ ± 0.63
Extract	39.08 ^b^ ± 2.08	355.33 ^b^ ± 1.33	74.06 ^b^ ± 1.10

Different letters in the same column indicate statistical difference using the Tukey test (*p* ≤ 0.05).

**Table 2:** Microcapsule characterization.

Parameters	MP	ME
AT (mg/100 g)	77.15 ^a^ ± 2.17	31.23 ^b^ ± 1.02
CFT (mg/100 g)	492.48 ^a^ ± 2.47	263.07 ^b^ ± 2.04
DPPH (%)	85.82 ^a^ ± 064	59.29 ^b^ ± 1.50
EE (%)	71.96 ^a^ ± 6.84	68.35 ^a^ ± 4.80
RTA (%)	85.71 ^a^ ± 4.47	80.14 ^a^ ± 6.01

Different letters on the same line indicate statistical difference using the Tukey test (*p* ≤ 0.05).

**Table 3:** Color parameters.

		Treatments	
Parameters		MP	ME
L*	L*_0_	55.06 ^a^ ± 0.1	61.11 ^b^ ± 0.12
	L*_f_	54.41 ^b^ ± 0.23	64.27 ^a^ ± 0.02
a*	a*_0_	16.12 ^b^ ± 0.11	24.32 ^b^ ± 0.04
	a*_f_	21.93 ^a^ ± 0.23	34.63 ^a^ ± 0.09
b*	b*_0_	1.20 ^a^ ± 0.02	7.27 ^a^ ± 0.09
	b*_f_	−0.77 ^b^ ± 0.02	5.69 ^b^ ± 0.02
C*	C*_0_	16.26 ^b^ ± 0.05	25.37 ^b^ ± 0.09
	C*_f_	21.94 ^a^ ± 0.23	35.09 ^a^ ± 0.09
H	h_0_	4.27 ^b^ ± 0.05	16.66 ^a^ ± 0.06
	h_f_	357.99 ^a^ ± 0.06	9.32 ^b^ ± 0.04
ΔE*	ΔE*	6.25 ± 0.08	10.89 ± 0.13

Different letters in the same column indicate statistical difference for each parameter at the initial and final evaluation using the Tukey test (*p* ≤ 0.05).

## Data Availability

The datasets supporting the conclusions of this article are included within the manuscript.
